# Supplementary figures and images for: Characterization and automatic classification of preterm and term uterine records
Source: PLoS One. 2018 Aug 28;13(8):e0202125. doi: 10.1371/journal.pone.0202125 (PMC6112643; doi:10.1371/journal.pone.0202125)

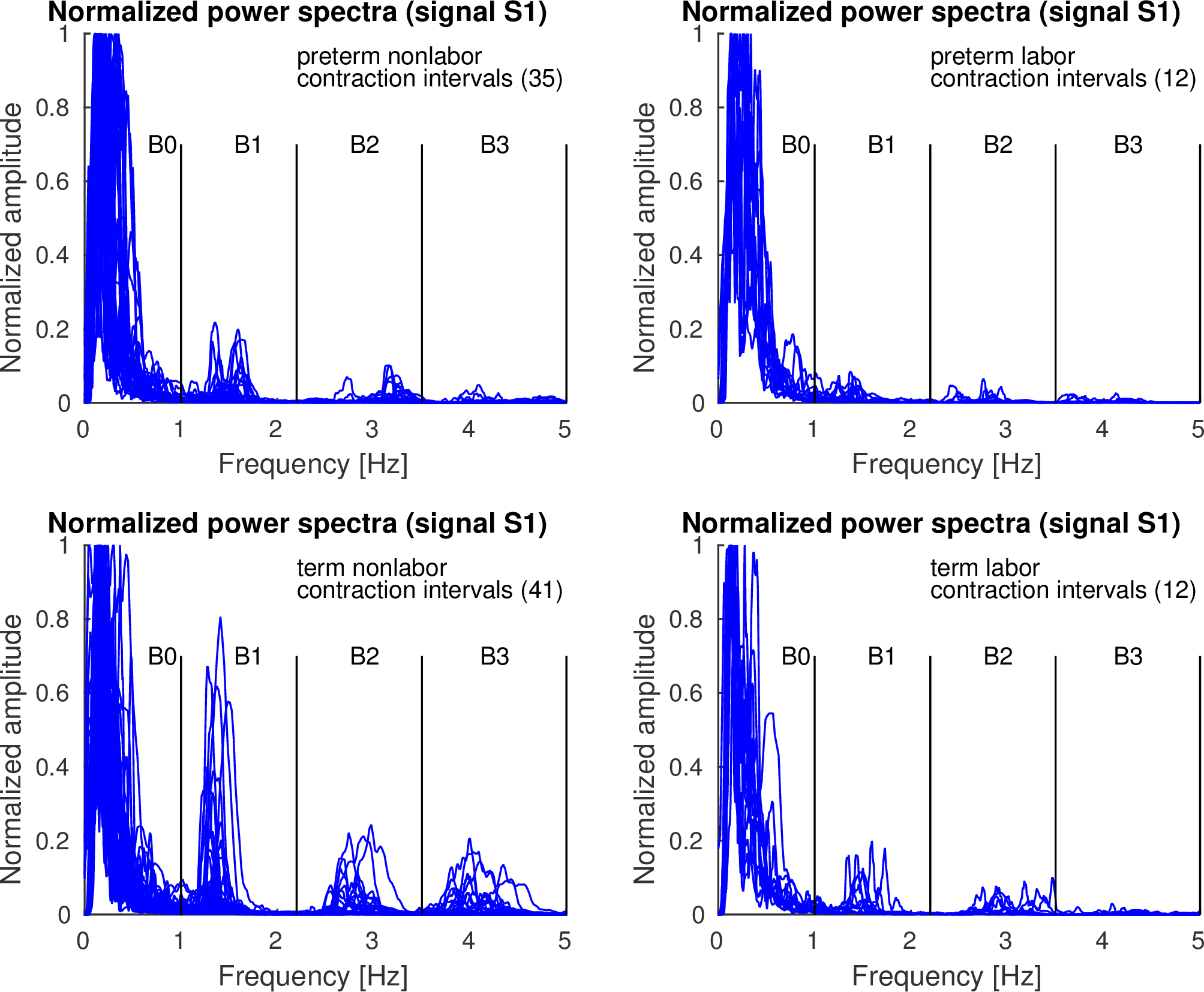

Supplement: S1 Fig — (TIF) [file pone.0202125.s001.tif]

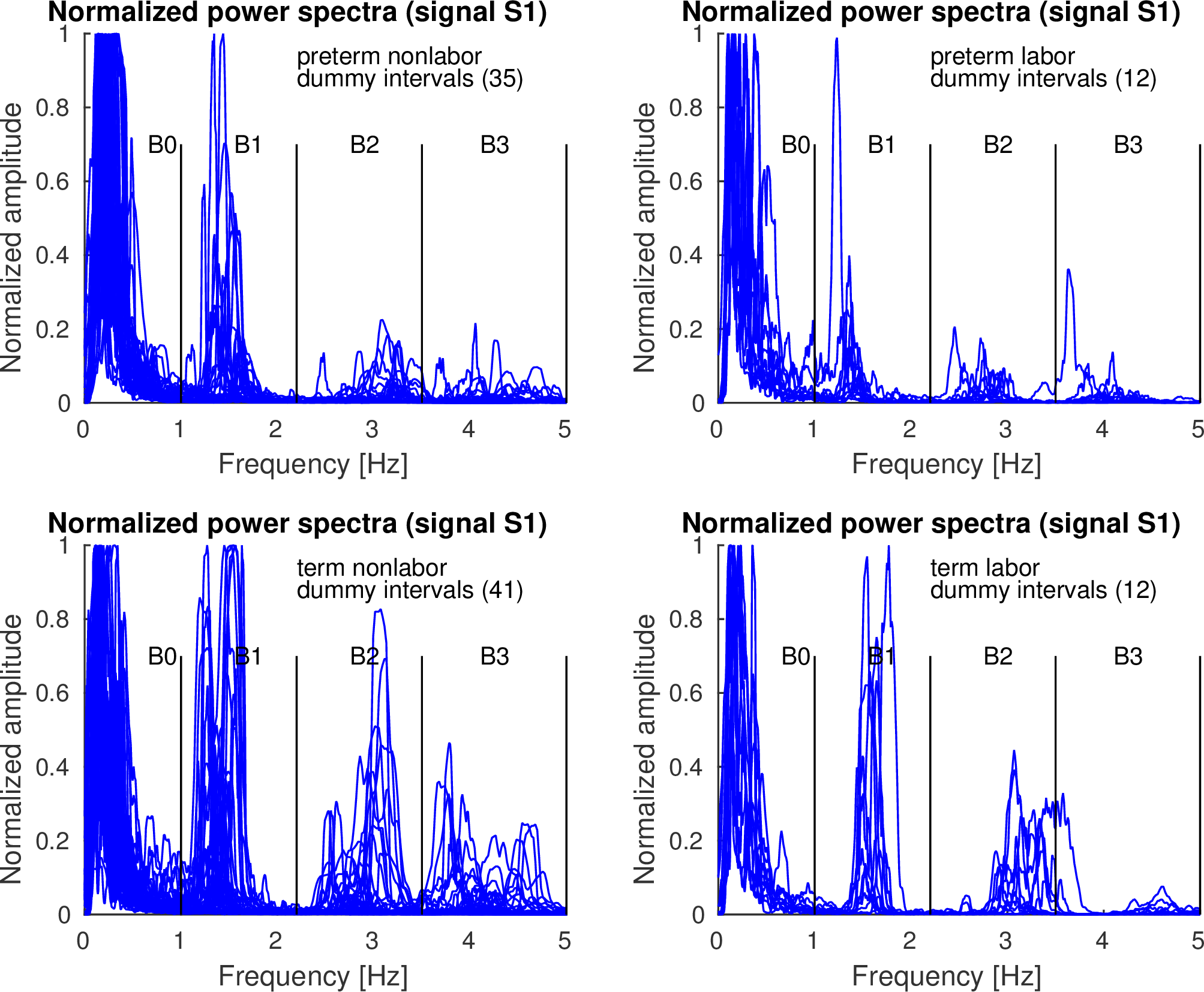

Supplement: S2 Fig — (TIF) [file pone.0202125.s002.tif]

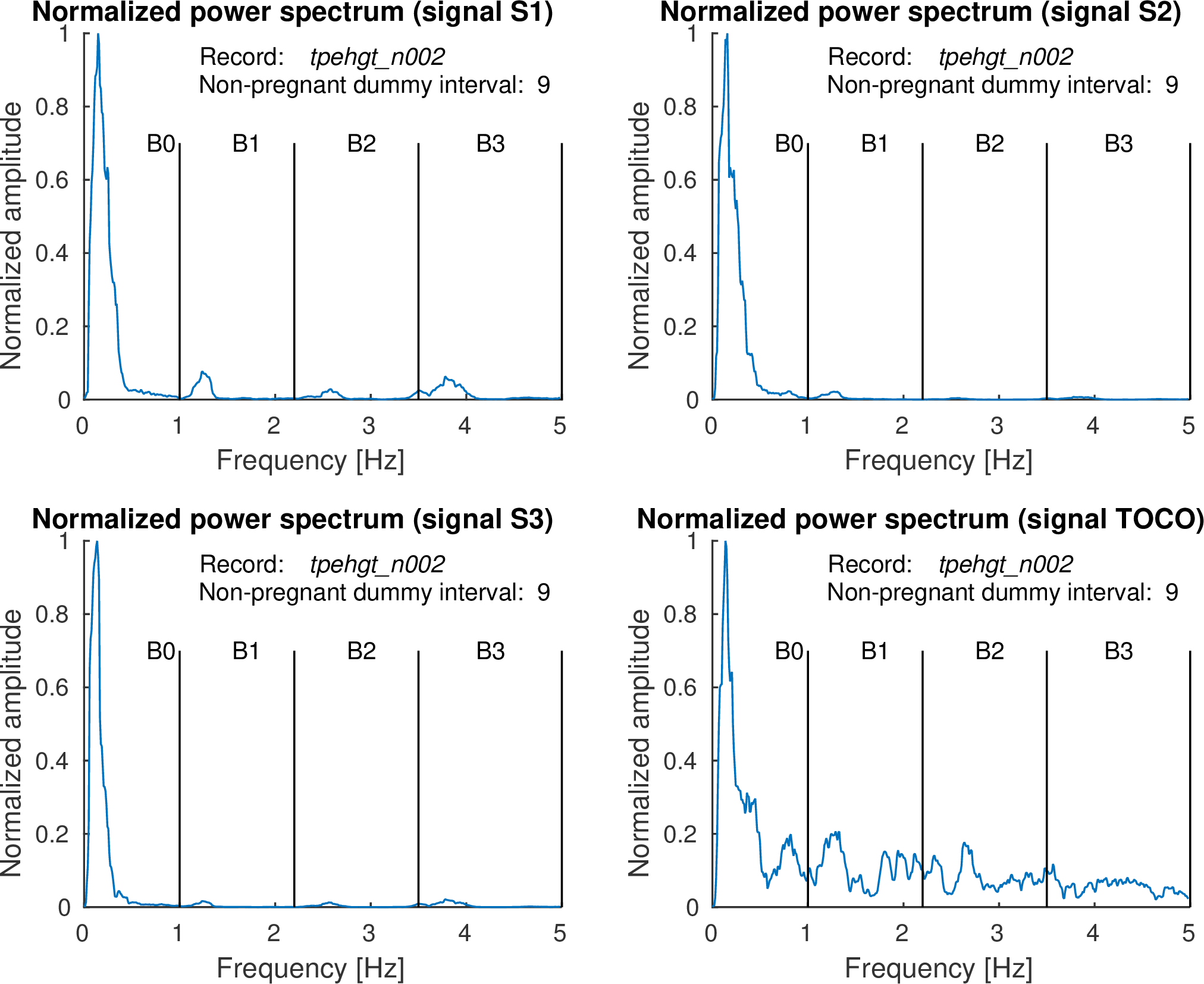

Supplement: S3 Fig — (TIF) [file pone.0202125.s003.tif]

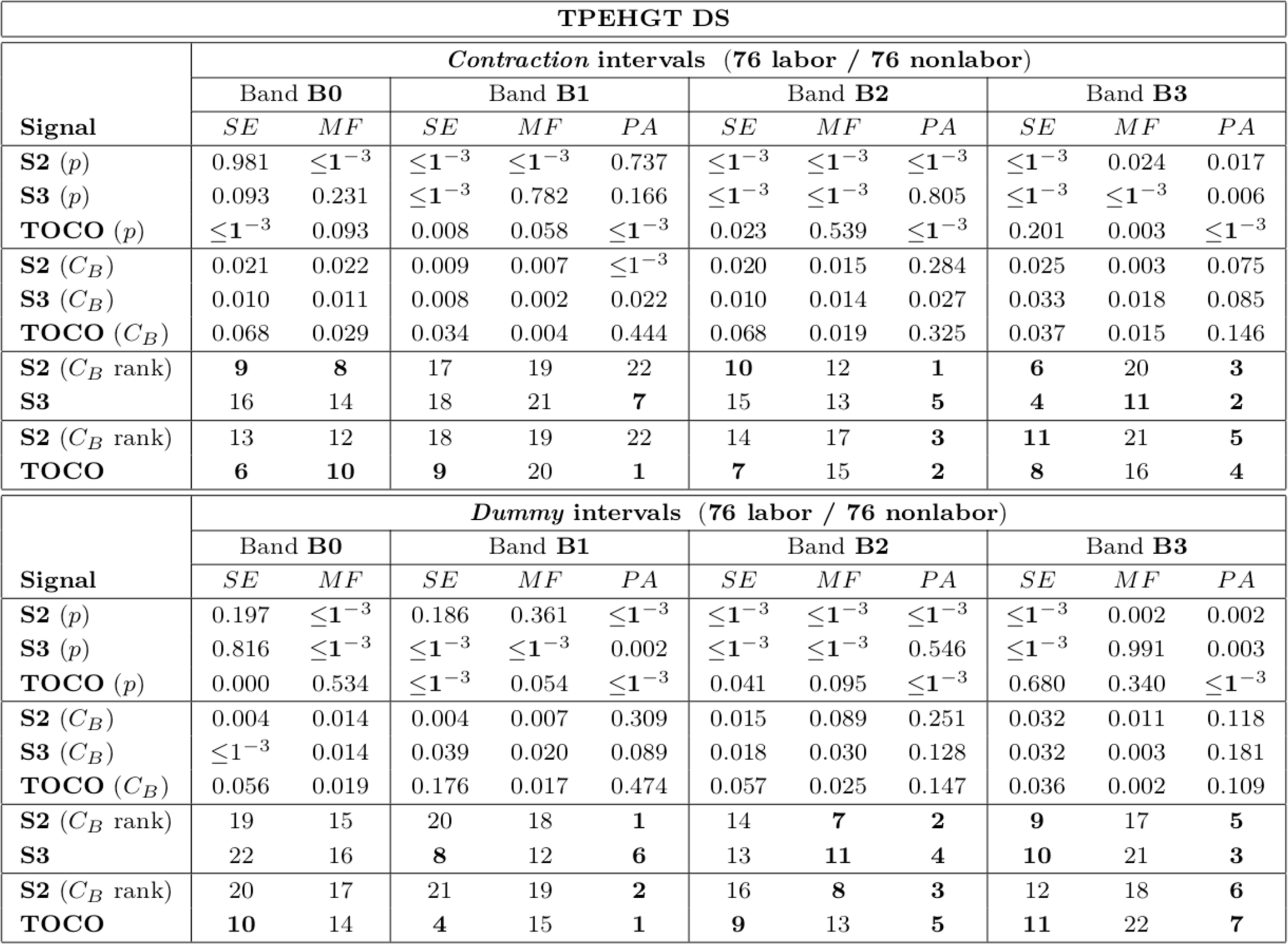

Supplement: S4 Fig — Those p values ≤1−3 and ranks of the first 11 features according to CB per group of signals are in bold. (TIF) [file pone.0202125.s004.tif]

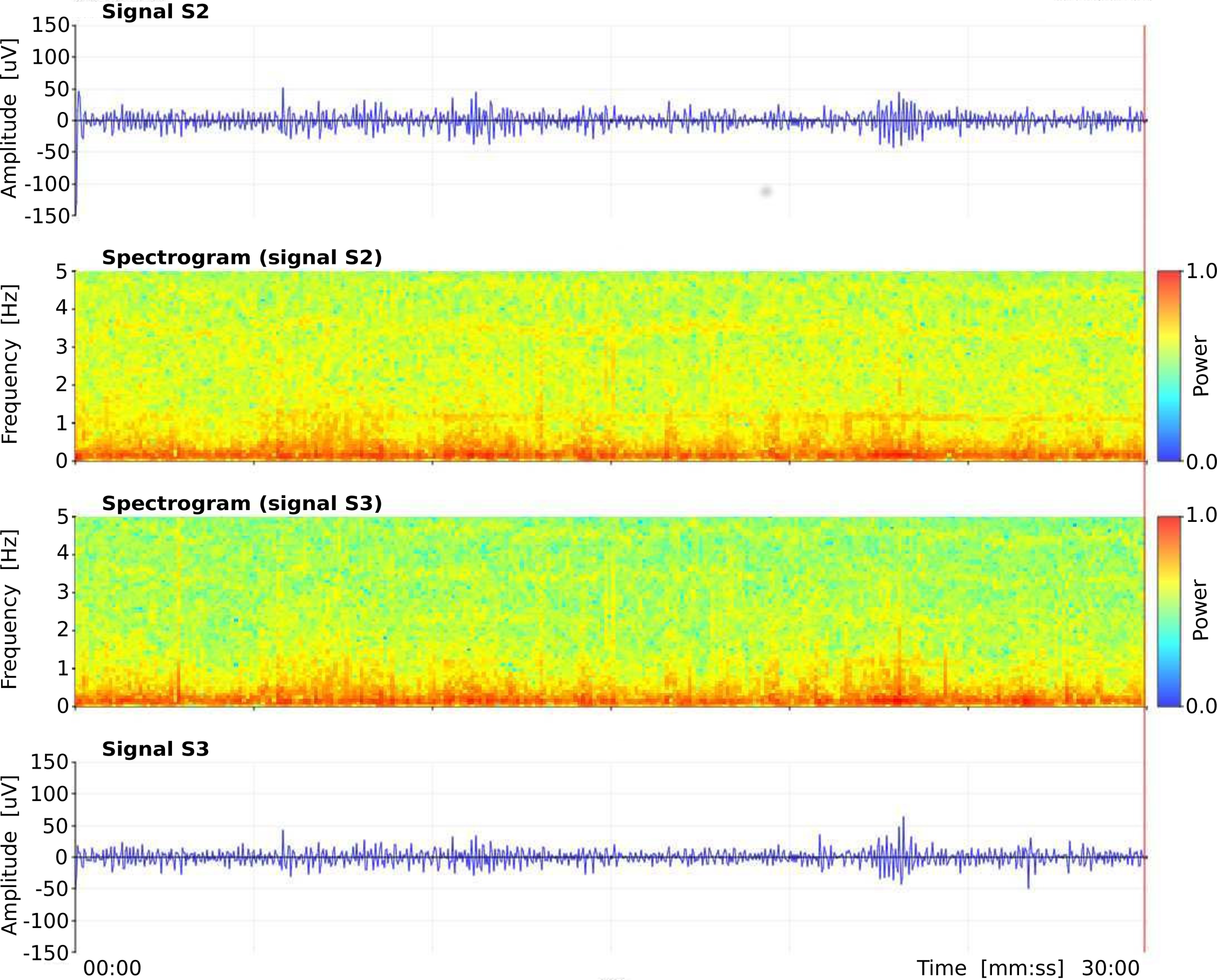

Supplement: S5 Fig — From top to bottom: EHG signal S2, spectrogram (0.0-5.0 Hz) of EHG signal S2, spectrogram (0.0-5.0 Hz) of EHG signal S3, EHG signal S3. (TIF) [file pone.0202125.s005.tif]

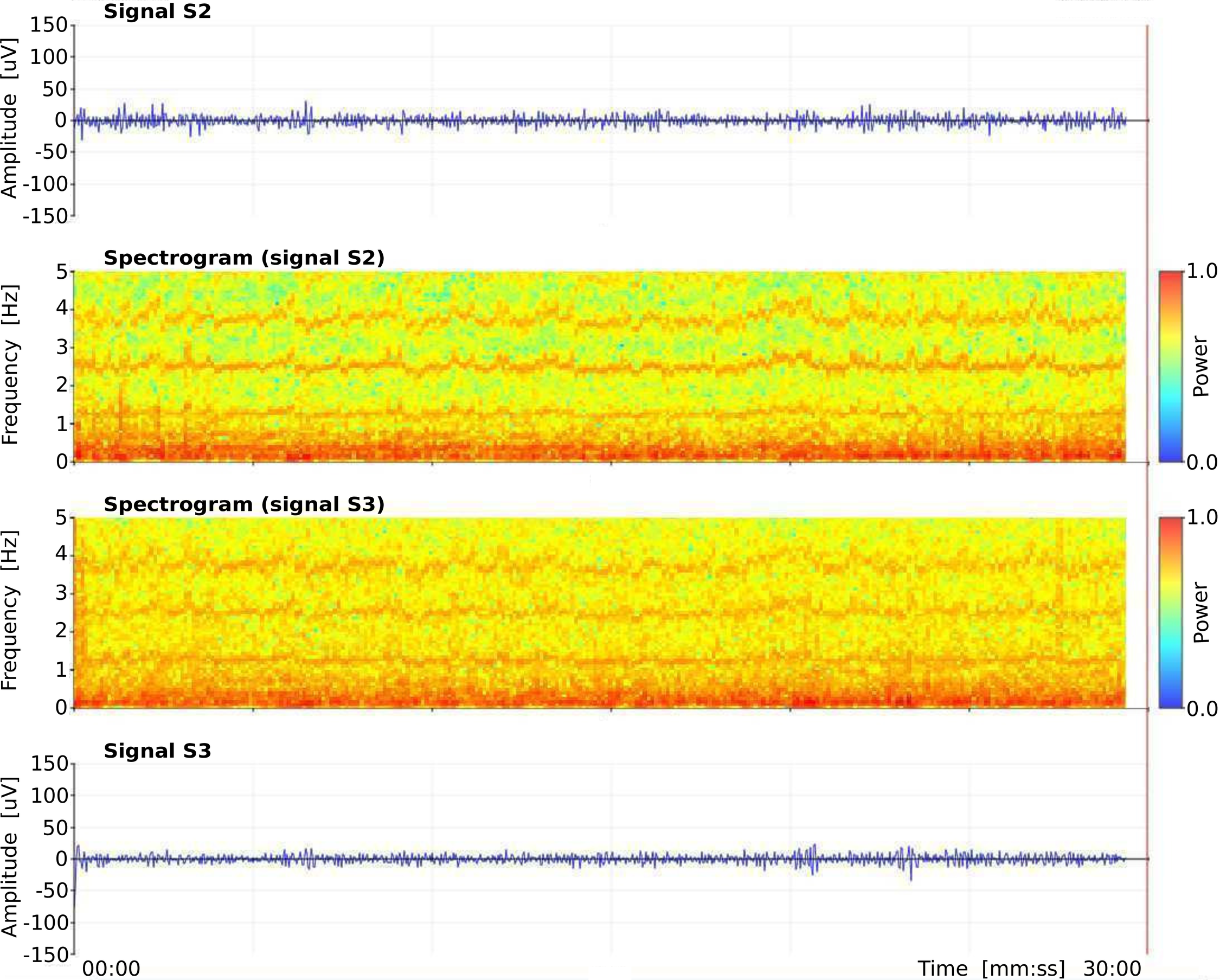

Supplement: S6 Fig — From top to bottom: EHG signal S2, spectrogram (0.0-5.0 Hz) of EHG signal S2, spectrogram (0.0-5.0 Hz) of EHG signal S3, EHG signal S3. (TIF) [file pone.0202125.s006.tif]
